# Supplementary material for: Socioeconomic status and ADL disability of the older adults: Cumulative health effects, social outcomes and impact mechanisms
Source: PLoS One. 2022 Feb 10;17(2):e0262808. doi: 10.1371/journal.pone.0262808 (PMC8830695; doi:10.1371/journal.pone.0262808)
Supplement: Attachment — Submitted filename: Response to Reviewers.docx [file pone.0262808.s001.docx]

**Revised**

**Reviewer #2:**

**Questions** 1. Absract; Great improvement. Well summarised and precise. Amendments noted especially on the last part of the abstract.
Introduction; It clearly shows gaps in literature that the research intends to fill-in. i however suggest that researchers use the 3rd party voice. They should avoid the 'we' aspect but to use the term 'researchers'.

**Revised:** Thank you for your comments and suggestions. According to your suggestion, we have replaced and deleted the "we" in the article and expressed it in the 3rd party voice.Please refer to the text for details, thank you!

**Questions** 2. Discussion; It sounds more like literature review with the focus on the gaps that need further studies. This part need revisiting. There is need to discuss findings of this particular research, then 'marry' the finding with literature, show the gap that the study has filled-in (new theory or knowledge). By end of discussion, the reader develops an idea of what is coming in the conclusion part of the research. Revisiting this part is needed.
**Revised:** Thank you for your comments and suggestions. According to your suggestion, we have made important adjustments to the discussion part to reflect the important conclusion contribution of this study and compared it with the existing research conclusions. So as to extract the advantages and limitations of this study.The specific amendments are as follows:

“Socioeconomic status is a comprehensive indicator of individual social participation and performance, and health risk is one of the most critical risks faced by individuals in their whole life. The results of this study demonstrate that the primary intermediary path of the impact of SES on the disability of the elderly is through health shock. Attribute to different levels of SES, there is remarkable group inequality of health shock among the elderly of different regions, and thus resulting in inequality of the degree of disability. In the research field of health status of the elderly, more and more researchers show their interest on the topics of situations of the elderly after serious diseases [2,49], in another word, the disability status. Therefore, this study is a contribution to this topic. This study reveals that SES is one of the important factors that affecting the incidence of serious disease of the elderly. The reasons are: from the perspective of individual function, disability is an inevitable outcome of the decline of various physical functions; from the social perspective, due to the influence of SES factors [50-52], the loss of physical function of the elderly is not only subject to the laws of general physical function, but also subject to the influence of their own social environment, such as differences in living habits and behavior norms brought by the differences of knowledge level, labor intensity before retirement and income [53-55]. And then, caused by the differences in daily living habits and behavior norms, inequality of health risks of elderly occurs. For example, the elderly of low education level are inclined to have more occurrence of bad habits, unhealthy eating and less exercise [56]; The elderly of low income are restricted by their own fiscal capacity, are tend to be short of healthy habits and behavior norms [51-52]. Consequently, they have much more high like hood to suffer from serious diseases than elderly of higher income levels.

As a summary, there are direct and indirect effects of SES on the risk of ADL disability of elderly. The direct benefits perform as low possibility of individual improvement or low accessibility to cares after encountering ADL disability; The indirect effects are mainly presented as the increased prevalence of individual serious diseases. Therefore, it is necessary to implement targeted treatment in combination with existing medical services or social services when considering policy intervention for the disabled elderly. From the existing studies of SES and ADL of the elderly, Lee et al. [57] demonstrate that multiple socioeconomic risks have a combined effect on cognitive impairment in old adults. Also, via the analysis of correlation between SES and various vulnerability components, Franse et al. [58] stated that inequality of vulnerability and vulnerability components exists due to unequal SES, and the number of individual morbid diseases is an important factor to explain the inequality of vulnerability of SES. These studies all illustrate that there is significant correlation between SES and individual health. Furthermore, from the relevant research of China, it is evidenced that SES that mainly evaluated by wealth, income and education has imposed significant impacts on residents' physical function. There are huge differences of functional health among the elderly in China due to unequal SES. Although this difference is more reflected by the decline of IADL, it is basically cased by the difference of education level [59-60]. In addition, high income was related to better IADL functioning but had no effect on the rate of change in IADL. High education was not associated with the baseline level or the rate of change in ADL score [61]. Dai et al. [62] also suggest that low SES may have a negative impact on the physical function of the elderly. This study further confirms that SES has a significant impact on the ADL disability of the elderly. Especially, it is evident of the reduction effect of low economic income and education level on the ADL of the elderly. However, compared with the existing research, the conclusion of this study is drawn based on the reality that disability risk is caused by health deterioration rather than the superficial causes of disability risk [2,49] Therefore, the findings of this study is an extension of the existing research which deepened into both of the direct and indirect effects path. This study contributes to the understanding that the impact of SES on ADL disability of the elderly not only comes from the direct effects from income and education, but also comes from the indirect effect of lower SES on the increase of health risk, which subsequently transmitted to the ADL of the elderly.

Therefore, it is necessary to adjust social and economic security policies in parallel with targeted treatment that based on existing medical services or social services, to improve economic security and optimize preventive health care measures for the elderly at the same time. Thus, this study also further enriched social research perspective that concerning ADL disability of the elderly, and provided solid foundation for formulating treatment and prevention policies for ADL disability of the elderly from the perspective of SES in the future.

In addition, the main content of this study is to investigate the logical relationship between SES and residents' ADL injury, and also focus on the impacts on disability that imposed by the cumulative effect of health. However, in the selection of multi-dimensional indicators of SES, due to the limitation of the macro survey database that we were not able to effectively match the onset time and duration of different diseases, the cumulative effect of health in this study is restricted to the statistics of health outcomes at the survey time point, which might affect the estimation results of effects of SES on health shock and ADL disability to a certain extent. This is also one of the main research deficiencies of this study. ”

**Questions** 3. Conclusion; Conclusion has improved. What is left is grammer. When concluding, one is bringing out one is bringing out finding of research thus no room for futuristic language as is in this case; ....... the rate of ADL disability will be lower whilst the rate of severe illness is positively affecting the role of ADL disability.... .

Vocabululary like 'obvious, seems' should be avoided. Conclusion should not base on assumptions but on empirical evidence of the research findings. *The is need to revisit line 5 of conclusion to the last sentence of the same conclusion and work on grammer.
Otherwise, the paper has greatly improved, there is evidence of amendments based on previous review.
**Revised:** Thank you for your comments and suggestions. According to your suggestion,we adjusted the "obvious, seems" used in the expression of the article , and we adjusted the grammar content related to the conclusion as follows:

“Based on the panel data of three periods of the CHARLS survey, this study empirically estimated the impact of SES on the risk of ADL disability risk of older adults, by using ologit regression and path analysis with health shock as mediator variable. The main findings are SES does impose significant impact on the ADL disability of older adults. In details, economic condition (income) plays dominant role, and there are significant differences among the urban, urban-rural fringe and rural older adults. Moreover, the various factors of health shock have significant and positive effects on the disability rate of older adults, and the effects are robust among urban, urban-rural fringe and rural areas. More specifically, the rate of ADL disability would be lower if physical pain is not felt, while the rate of ADL disability would be higher if the rate of severe illness is high. From the respect of the impacts of SES on the health of older adults, education and economic status are significant, yet group inequality is not observed.

The results of estimation of path effect suggest that there is significant group inequality in the path effect of SES on the ADL disability of older adults. Specifically, SES imposes positive impacts on the rate of non-pain and psychological depression of the urban older adults, while for the rural older adults, SES significantly affects the rate of non-pain, psychological depression, and ADL disability. Thus, the effecting path of SES on ADL disability is mainly based on the rate of severe illness, physical pain and sight.

At last, in the further expansion of this study, we can take the construction of indicators of disability inequality as the core target, to investigate the evolution track of disability inequality under the cumulative effect of different health levels and different disease categories. It would be more insightful to provide effective theoretical and empirical support for effective policy intervention.”
